# Genome-Wide Identification of GRAS Gene Family and Drought Response Analysis of DELLA Proteins in *Populus deltoides*

**DOI:** 10.3390/cimb48060541

**Published:** 2026-05-22

**Authors:** Changgeng Shang, Hu Huang, Yu Chen, Renying Zhuo, Hongsuo Shu, Zhengquan He

**Affiliations:** 1 Key Laboratory of Three Gorges Regional Plant Genetic & Germplasm Enhancement (CTGU), Biotechnology Research Center, China Three Gorges University, Yichang 443002, China; 15090903900@163.com; 2 State Key Laboratory of Tree Genetic and Breeding, Research Institute of Subtropical Forestry, Chinese Academy of Forestry, Hangzhou 311400, China; 15272978041@163.com (H.H.); zhuory@gmail.com (R.Z.); 3 Agricultural Technology Extension Centre of Dongtai, Yancheng 224200, China; chenyu199601201@163.com; 4 Sanmen Forestry Extension Station of Zhejiang, Taizhou 317100, China; shuhstz@126.com

**Keywords:** genome-wide analysis, GRAS genes, drought stress, DELLA proteins, *Populus deltoides*

## Abstract

The GRAS transcription factor family plays a pivotal role in plant stress adaptation, yet its systematic characterization and the underlying drought-responsive mechanisms remain poorly elucidated in *Populus deltoides*. Here, a genome-wide identification and analysis of GRAS genes in *P. deltoides* was performed, and a total of 92 family members were identified and classified into 12 distinct subfamilies through phylogenetic analysis. Evolutionary analysis revealed a high degree of conservation between the GRAS proteins of *P. deltoides* and those of *Arabidopsis thaliana*, *Oryza sativa*, and *Solanum lycopersicum*. Genomic duplication events, including 90 segmental and 11 tandem duplications, were identified as the primary drivers of GRAS family expansion. Promoter *cis*-element analysis uncovered an enrichment of stress-responsive elements (MBS, ABRE) and phytohormone-related motifs (e.g., TATC-box). Transcriptomic profiling further revealed distinct drought-inducible expression patterns of *GRAS* genes: *PdeGRAS49* exhibited rapid upregulation at the early stage of drought exposure (1–3 h), whereas DELLA subfamily members *PdeGRAS51* and *PdeGRAS59* reached their expression peaks at 6–9 h, and *PdeGRAS34* and *PdeGRAS77* maintained sustained activation throughout 12–24 h. Moreover, the drought-inducible expression patterns of five *DELLA* genes were confirmed by qRT-PCR validation. Collectively, this study provides crucial genomic insights into the GRAS family and valuable candidate gene resources, which lay a foundation for molecular breeding of drought-tolerant *P. deltoides* cultivars via manipulating GRAS-mediated regulatory mechanisms.

## 1. Introduction

Global warming has intensified the frequency and severity of drought events, posing a critical threat to plant survival, growth, and agricultural productivity worldwide [1,2,3]. Elucidating the molecular mechanisms underlying plant drought responses is therefore crucial for developing strategies to enhance stress tolerance in economically and ecologically important species. Abscisic acid (ABA) is a key plant hormone that regulates the response of plants to drought stress. The ABA response includes stomatal closure, gene expression, accumulation of osmoprotectants and stress proteins. Under drought stress, plants use both ABA-dependent and ABA-independent signaling pathways to mediate intracellular signaling and the expression of stress-related genes, thereby enabling them to survive in arid environments [4].

*Populus* species, valued for their rapid growth and broad ecological adaptability, serve as cornerstones for global afforestation and bioenergy production [5]. However, increasing aridity in major cultivation zones has severely constrained their productivity, diminishing both economic returns and critical ecosystem services [5,6]. Among these, *Populus deltoides* is particularly notable for its exceptional economic value, providing raw materials for timber and biofuels while playing a key role in riparian stabilization. Paradoxically, its high productivity is inherently linked to high water consumption, making the species highly sensitive to drought [7]. As climate change increases the frequency of extreme water-deficit events, a deep understanding of the molecular mechanisms that govern the balance between growth and stress responses in *P. deltoides* has become a paramount research priority [8].

The GRAS gene family, a plant-specific and functionally important group of transcription factors, plays a critical role in plant growth and development, hormone signaling, and stress responses [9]. Genome-wide identification of the GRAS family has been completed across various model and crop species, providing a foundation for comparative genomics. Key identified counts include *Arabidopsis thaliana* (32–34 members), rice (*Oryza sativa*, 57–60 members), soybean (*Glycine max*, 117 members), wheat (*Triticum aestivum*, 177 members), and apples (*Malus domestica*, 127 members) [10,11,12,13,14]. In soybean, specific members like GmGRAS37 have already been confirmed to significantly improve drought and salt tolerance [12]. While their roles have been characterized in some model species, a comprehensive genome-wide analysis of the GRAS gene family in *P. deltoides* and its specific contribution to responding to drought stress is currently lacking. Based on sequence and functional characteristics, the GRAS family is divided into several subfamilies, including DELLA, HAM, LAS, PAT1, SCR, SCL3, LISCL, and SHR [15,16]. Each subfamily has distinct roles in specific physiological processes. For example, the SCR and SHR subfamilies are involved in the formation of the SCR/SHR complex, which regulates root and shoot branching during plant development. The DELLA subfamily primarily functions as a repressor in the gibberellic acid (GA) signaling pathway, modulating plant growth and development [17]. DELLA proteins also enhance drought tolerance by regulating GA signaling [18]. Through their involvement in GA signaling and interactions with other hormone pathways, DELLA genes play a pivotal role in plant responses to drought stress [19]. These mechanisms not only help plants maintain growth under drought conditions but also improve their drought resistance. Thus, in-depth research on DELLA genes and their associated signaling pathways is essential for developing new drought-tolerant plant varieties [20].

This study presents the first comprehensive analysis of the GRAS gene family in *P. deltoides*, identifying a total of 92 GRAS members and classifying them into 12 subfamilies. We systematically identified and characterized their gene structures and *cis*-acting elements in promoters, as well as gene duplication, synteny, and phylogenetic relationships. Additionally, a detailed expression profiling of the DELLA subfamily within the PdeGRAS family under PEG-simulated drought stress was conducted. These findings provide a foundation for further research into the functional roles and regulatory mechanisms of *PdeGRAS* genes under drought conditions and offer a potential candidate gene, *PdeGRAS77*, for breeding drought-tolerant plant varieties.

## 2. Materials and Methods

### 2.1. Plant Growth Conditions and PEG-Simulated Drought Treatment

Three-week-old *P. deltoides* seedlings, propagated through micropropagation, were initially cultured in 1/2 Hoagland nutrient solution for 7 days under controlled conditions in an artificial climate chamber (25 °C, 16 h of light, and 8 h of darkness) [21]. Following this, the plants were exposed to simulated drought stress by treatment with a 10% PEG6000 solution for varying durations: 0 h, 1 h, 3 h, 6 h, 9 h, 12 h, and 24 h. A control group was maintained under identical conditions but irrigated with tap water instead of PEG6000. After treatment, root, stem, and leaf samples were collected in triplicate to minimize experimental errors.

### 2.2. Identification of the GRAS Family Genes in P. deltoides

Detailed genomic information of *P. deltoides* was retrieved from the Phytozome V13 database (https://phytozome-next.jgi.doe.gov/, accessed on 21 January 2025) to facilitate the subsequent identification of GRAS transcription factors [22]. The hidden Markov model (HMM) of the GRAS domain family (PF03514) was downloaded from the Pfam website (http://pfam.xfam.org/, accessed on 21 January 2025). Local HMMsearch was performed using an E-value threshold of 1 × 10^−10^ to identify all family members containing conserved GRAS domains. The relative molecular weight (MW) and isoelectric point (pI) of each GRAS family member were predicted and calculated using the ExPASy online tool (https://web.expasy.org/compute_pi/, accessed on 21 January 2025). Additionally, subcellular localization of the GRAS family members was predicted using the Plant-mPLoc online platform (http://www.csbio.sjtu.edu.cn/bioinf/plant-multi/, accessed on 25 January 2025).

### 2.3. Chromosomal Location and Gene Duplication of PdeGRASs

Homologous sequences of *GRAS* genes in *P. deltoides* were aligned using the local BLAST 2.15.0 program to generate duplicate gene pairs. The collinearity relationships of *GRAS* genes were analyzed using MCScanX 1.0.0 [23]. Gene pairs with both alignment coverage and similarity greater than 75% were considered to have undergone potential gene duplication events. Tandem duplications were defined as repeated genes with fewer than one intervening gene on a single chromosome; otherwise, they were classified as segmental duplications in this study. The distribution of *PdeGRAS* genes across different chromosomes of *P. deltoides* was visualized using TBtools v2.441 [24].

### 2.4. Phylogenetic Analysis of PdeGRASs

The complete protein sequences of *A. thaliana*, *O. sativa*, and *Solanum lycopersicum* were downloaded from the Phytozome V13 database. Gene IDs and corresponding full-length protein sequences of GRAS transcription factors from these species were extracted and screened using the Local HMMsearch. In addition, 92 *GRAS* genes identified in *P. deltoides* were included in the analysis. Multiple sequence alignment was performed using MUSCLE, and a phylogenetic tree was constructed using RaxML 8.2.13 software with the PROTGAMMAJTT model. To assess the robustness of the tree, bootstrap analysis was performed with 1000 replicates using the default parameters. The phylogenetic tree was visualized using the ChiPlot (https://www.chiplot.online/tvbot.html, accessed on 12 March 2025) online tool.

### 2.5. Gene Structure and Protein Motifs Analysis

The exon–intron structures of each *PdeGRAS* gene were analyzed and visualized using their respective GFF annotation files with TBtools software v2.441. Conserved motifs in each PdeGRAS protein were predicted using the Multiple Em for Motif Elicitation (MEME) online tool (http://meme-suite.org/tools/meme, accessed on 8 May 2025) under default parameters, with the maximum number of motifs set to 10 [25,26]. The predicted motifs were subsequently visualized using GSDS 2.0 software [27,28].

### 2.6. Cis-Acting Element Analysis in PdeGRASs Promoters

The ATG upstream 2000 bp sequences of GRAS family members in *P. deltoides* were extracted using TBtools v2.441 and submitted to the PlantCARE online tool (http://bioinformatics.psb.ugent.be/webtools/plantcare/html/, accessed on 21 June 2025) for the prediction of *cis*-regulatory elements in the promoter regions. The identified *cis*-regulatory elements were statistically analyzed and visualized using GSDS 2.0 software.

### 2.7. RNA Extraction and qRT-PCR Analysis

Total RNA was extracted from root, stem, and leaf samples of *P. deltoides* seedlings using the RNAprep Pure Plant Plus Kit (TIANGEN, Beijing, China). Eight-week-old wild-type *P. deltoides* ‘Danhong’ seedlings were exposed to 10% PEG6000- simulated drought stress for 0–24 h and subjected to RNA sequencing (RNA seq) analysis using an Illumina Novaseq6000. Raw data were processed on BMKCloud (www.biocloud.net, accessed on 28 June 2025). The dataset of RNA-seq was deposited in the National Genomics Data Center, with the BioProject accession number PRJCA044951. First-strand cDNA synthesis was performed using the PrimeScript™ RT Master Mix (TaKaRa, Dalian, China). Quantitative primers were designed using TBtools v2.441, and qRT-PCR was conducted with the 2 × Q3 SYBR qPCR Master Mix (TOLOBIO, Shanghai, China) on a 7300 Real-Time PCR System (Thermo Fisher, Carlsbad, CA, USA). Technical replicates were conducted three times for each sample. Relative expression levels were quantified using the 2^−ΔΔCT^ method with the actin gene serving as a reference [29,30]. Primers used are listed in Table S1. The relative expression data obtained from qRT-PCR were used to generate heatmaps in TBtools v2.441.

### 2.8. Protein–Protein Interaction (PPI) Network Analysis and 3D Structure Modeling

To systematically characterize molecular interactions among DELLA subfamily members, a comprehensive in silico analysis was performed on five key PdeGRAS proteins (PdeGRAS29, PdeGRAS51, PdeGRAS59, PdeGRAS77, PdeGRAS86). The STRING database (v12.0) predicted functional associations using high-confidence parameters (interaction score > 0.7), followed by structural modeling through the SWISS-MODEL workspace with global quality estimates (GMQE > 0.6) and sequence identity thresholds (>40%). This integrated bioinformatics approach generated both interaction networks and validated 3D structural models, revealing potential dimerization interfaces and conserved domain architectures critical for DELLA protein functions [31,32].

### 2.9. Subcellular Localization

The full-length coding sequence (CDS) of *PdeGRAS77* was amplified (1302-PdeGRAS77-F and 1302-PdeGRAS77-R; Table S1) from drought-stressed leaf tissues. Homologous recombination was performed using Bgl II and Spe I restriction sites to ligate the ORF into the multiple cloning site of the pCAMBIA1302 binary vector, fusing the original GFP coding sequence while maintaining the CaMV 35S constitutive promoter. The recombinant plasmids were electroporated into *Agrobacterium tumefaciens* strain GV3101 (pSoup-p19) for subsequent transient transformation of *Nicotiana benthamiana* epidermal cells via syringe infiltration. Subcellular localization was confirmed by laser scanning confocal microscopy, detecting GFP fluorescence. *35S_pro_::D53-mCherry* was used as a nucleus marker [33].

### 2.10. Statistical Analysis

Data are expressed as the mean ± SE (*n* = 3) and were employed using Student’s *t*-test, * *p* < 0.05; ** *p* < 0.01. followed by subsequent analysis with GraphPad Prism 10.1.2 software.

## 3. Results

### 3.1. Identification and Classification of PdeGRAS Proteins in P. deltoides

Through HMMsearch screening and analysis, a total of 92 PdeGRAS family members were identified in the *P. deltoides* genome, which were designated as PdeGRAS1 to PdeGRAS92. Among these proteins, PdeGRAS83 was the longest, consisting of 1096 amino acids with a molecular weight of 125.53 kDa, while PdeGRAS31 was the shortest, containing only 147 amino acids and a molecular weight of 17.07 kDa. The isoelectric points (pI) of these proteins ranged from 4.74 (PdeGRAS32) to 9.63 (PdeGRAS30). Subcellular localization predictions revealed that 88 out of the 92 PdeGRAS proteins were localized in the nucleus, while the remaining four (PdeGRAS10, PdeGRAS27, PdeGRAS31, and PdeGRAS91) were predicted to be localized in the chloroplast or nucleus (Table S2).

### 3.2. Phylogenetic Analysis of GRAS Genes

**Figure 1:**
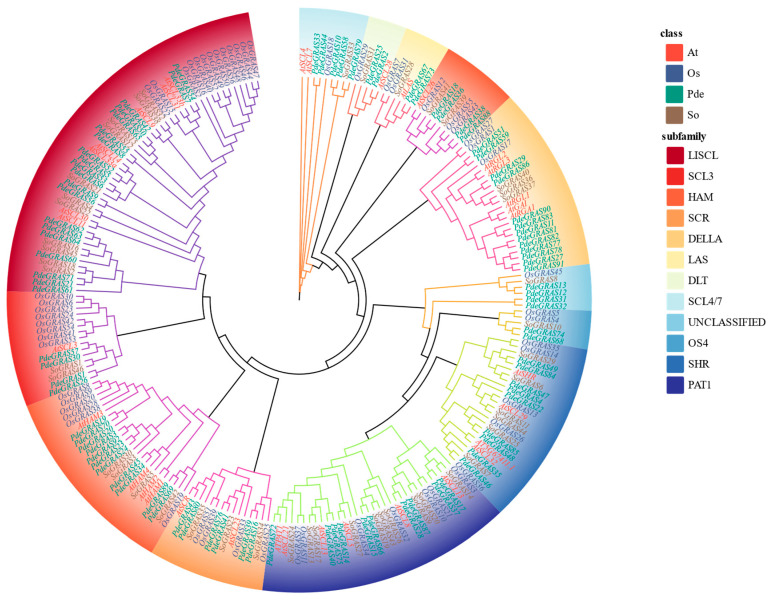
Phylogenetic tree of the GRAS family in *A. thaliana*, *O. sativa*, *P. deltoides*, and *S. lycopersicum*. The block of four different colors on the right denotes the GRAS family members corresponding to *A. thaliana*, *O. sativa*, *P. deltoides*, and *S. lycopersicum*. Branches corresponding to each subfamily are depicted in distinct colors, with those of members within the same subfamily shown in identical colors. The phylogenetic tree was constructed using the ML method with 1000 bootstrap replicates.

To better understand the evolutionary relationships and functional significance of GRAS proteins in *P. deltoides*, a maximum likelihood phylogenetic tree was constructed using GRAS proteins from *A. thaliana*, *O. sativa*, *P. deltoides*, and *S. lycopersicum*. A total of 34 *GRAS* genes from *A. thaliana*, 56 from *O. sativa*, 92 from *P. deltoides*, and 46 from *S. lycopersicum* were included in the analysis (Table S3). The phylogenetic tree was divided into 12 subfamilies (Figure 1). Among the 92 PdeGRAS members, 20 were classified into the LISCL subfamily, 4 into SCL3, 15 into HAM, 6 into SCR, 2 into LAS, 2 into DLT, 5 into SCL4/7, 2 into OS4, 9 into SHR, 10 into PAT1, and 13 into the DELLA subfamily [34]. Additionally, 4 PdeGRAS members could not be assigned to any specific subfamily.

### 3.3. Chromosomal Location of PdeGRAS Genes

**Figure 2:**
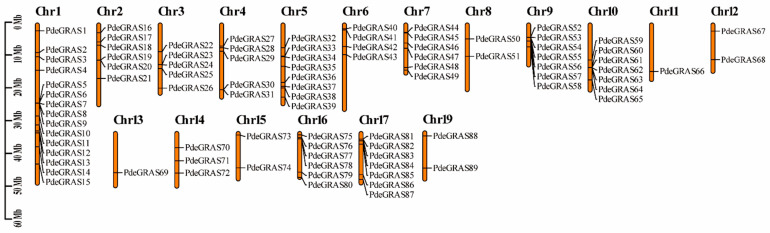
Chromosomal localization of PdeGRAS family members in *P. deltoides.* The vertical axis indicates chromosomal length. “Chr1” to “Chr19” represent the 19 chromosomes of *P. deltoides*, with black labels indicating the positions of the 89 *GRAS* genes on the respective chromosomes.

To investigate the chromosomal distribution of the 92 PdeGRAS family members in *P. deltoides*, the physical locations of all 92 genes across the chromosomes were analyzed (Figure 2). The results revealed that the 89 PdeGRAS members were distributed across 18 chromosomes. Notably, no *PdeGRAS* genes were identified on Chr18. Chr1 contained the highest number with 15 *PdeGRAS* genes (Table S4).

### 3.4. Gene Structure and Conserved Motif Analysis of PdeGRASs

A comprehensive analysis was performed to provide insights into the structural characteristics and conserved functions of the PdeGRAS family genes in *P. deltoides*. The results showed that the *PdeGRAS* genes in the same subfamily had similar gene structure and motifs, which were consistent with the results of the phylogenetic analysis (Figure S1). Every member had Motif 5, Motif 8, Motif 2, Motif 3 and Motif 7. Based on the analysis of the structure and conserved motifs of *PdeGRASs* in different subclades, it is clear that members within the same subclade are relatively conserved. The exon/intron gene structure plays a crucial role in the evolution of gene families. About 23 PdeGRAS family members were found to contain introns, and the remaining members showed structural similarities, implying the functions of genes in the same subclade may be similar.

### 3.5. Gene Duplication and Synteny Analysis of PdeGRASs

**Figure 3:**
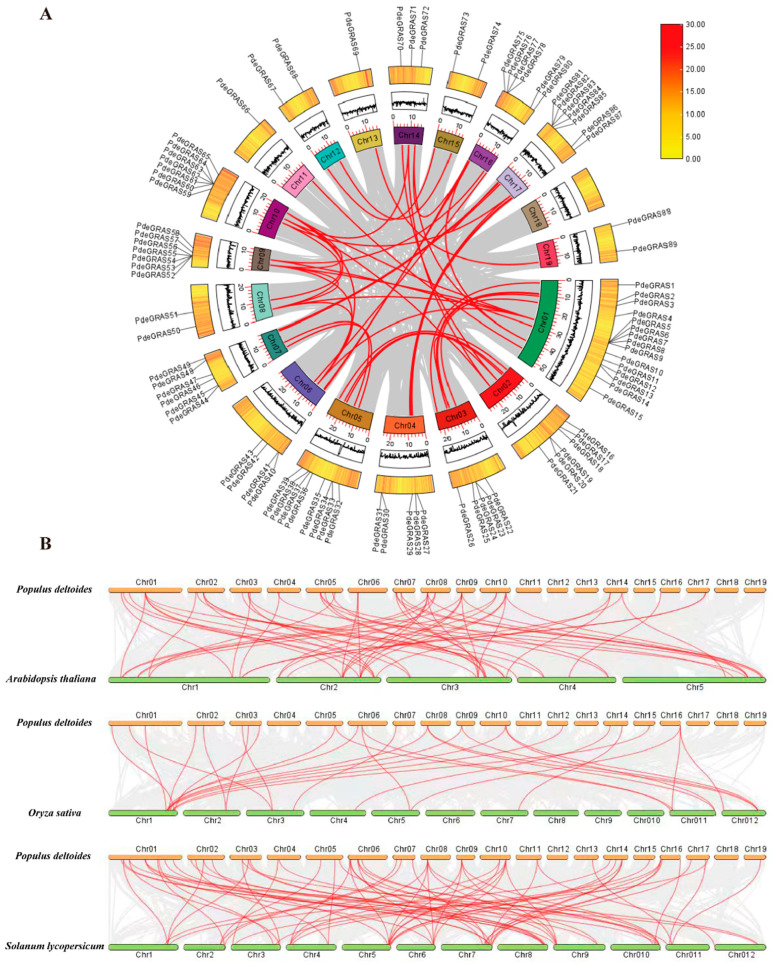
Co-linearity analysis of the *PdeGRAS* genes. (**A**) Intraspecific collinearity within *P. deltoides* was analyzed, with red lines and arcs indicating segmental duplication events. (**B**) Interspecific collinearity among *P. deltoides*, *A. thaliana*, *O. sativa*, and *S. lycopersicum* was examined. Gray lines represent all collinear gene pairs between *P. deltoides* and the other species, while red lines denote collinear GRAS gene pairs.

To investigate the evolutionary trajectories among PdeGRAS family members in *P. deltoides*, the gene duplication events within the PdeGRAS gene family were analyzed. As shown in Figure 3A, among the 92 PdeGRAS family members, 90 segmental duplication events and 11 tandem duplication gene pairs were identified (Table S5). These results suggest that segmental duplication has likely been a major driving force in the evolution of the PdeGRAS family members. To elucidate the evolutionary relationship between PdeGRASs and other GRASs, a collinearity analysis was performed comparing *A. thaliana*, *O. sativa*, and *S. lycopersicum*. The analysis confirmed that *P. deltoides* shared 57, 29, and 71 orthologous gene pairs with *A. thaliana*, *O. sativa*, and *S. lycopersicum*, respectively (Figure 3B). Interestingly, dicot plants (poplar, tomato, *Arabidopsis*) exhibited greater gene pairs compared with monocot plants (rice).

### 3.6. Cis-Acting Elements in the Promoters of PdeGRAS Genes

**Figure 4:**
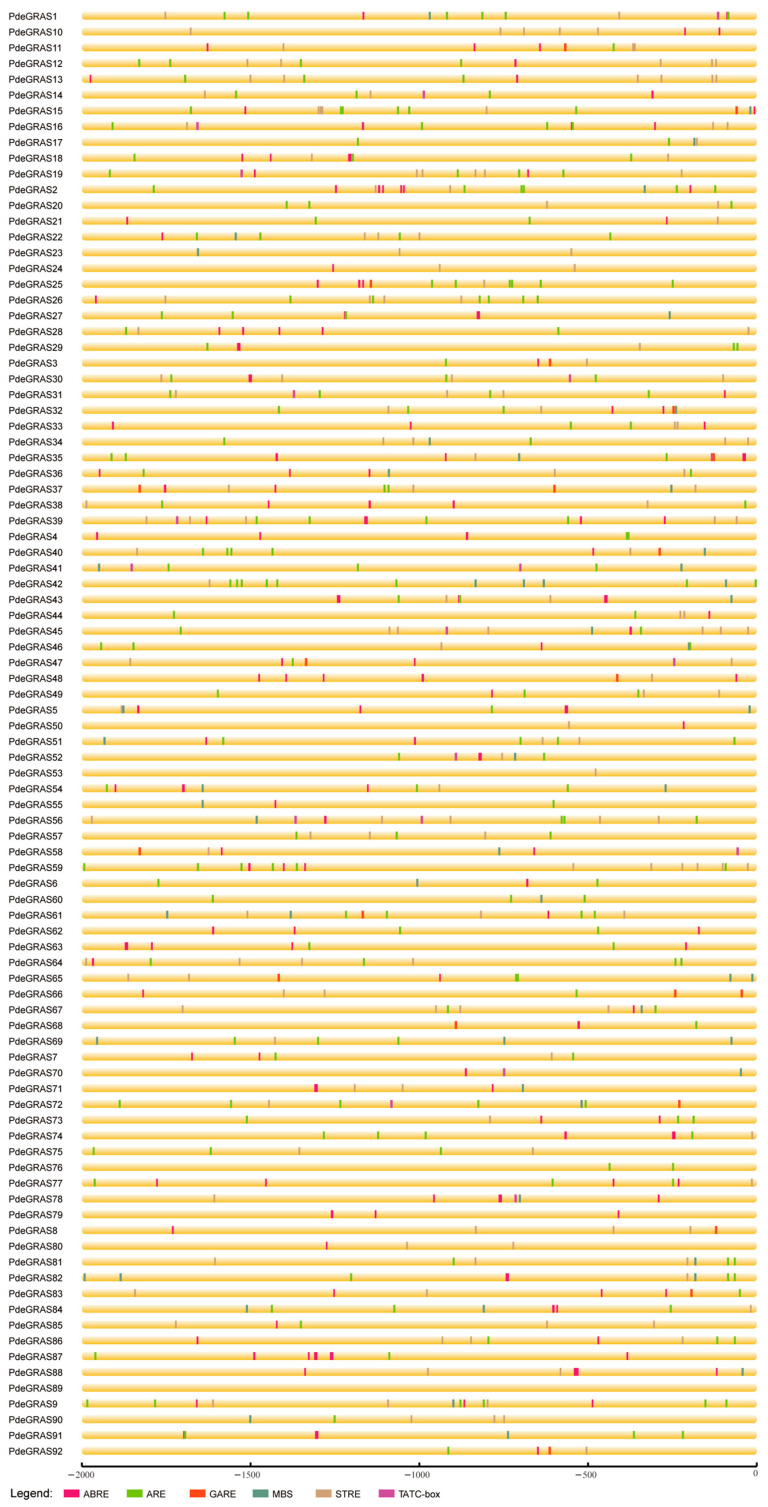
Distribution of *cis*-regulatory elements of *PdeGRAS* promoters. Rectangles with different colors indicate different *cis*-acting elements. ABRE represents ABA-responsive element, ARE represents anaerobic-responsive element, GARE represents gibberellin-responsive element, MBS represents MYB binding sites, STRE represents stress-responsive element, and TATC-box represents *cis*-acting element involved in gibberellin-responsiveness.

To analyze the *cis*-acting elements in the promoter regions of PdeGRAS family members, the 2000 bp upstream sequences of each *PdeGRAS* gene from the genome were extracted, and potential regulatory elements were predicted (Figure 4). The analysis revealed a variety of stress-responsive and hormone-responsive elements. Among these, elements associated with ABA-mediated osmotic stress signaling and drought stress response were widely distributed in the promoter regions of many PdeGRAS members: 76 members contained ABRE elements (ABA-responsive element), 77 members contained ARE elements (anaerobic-responsive element), 19 members contained GARE elements (gibberellin-responsive element), 42 members contained MBS elements (MYB binding sites), 77 members contained STRE elements (stress-responsive element), and 17 members contained TATC-box elements that participate in the gibberellin signaling pathway. Collectively, these findings indicate that the majority of PdeGRAS members are likely to be involved in the regulation of drought stress response.

### 3.7. Expression Profiling Analysis of PdeGRASs

**Figure 5:**
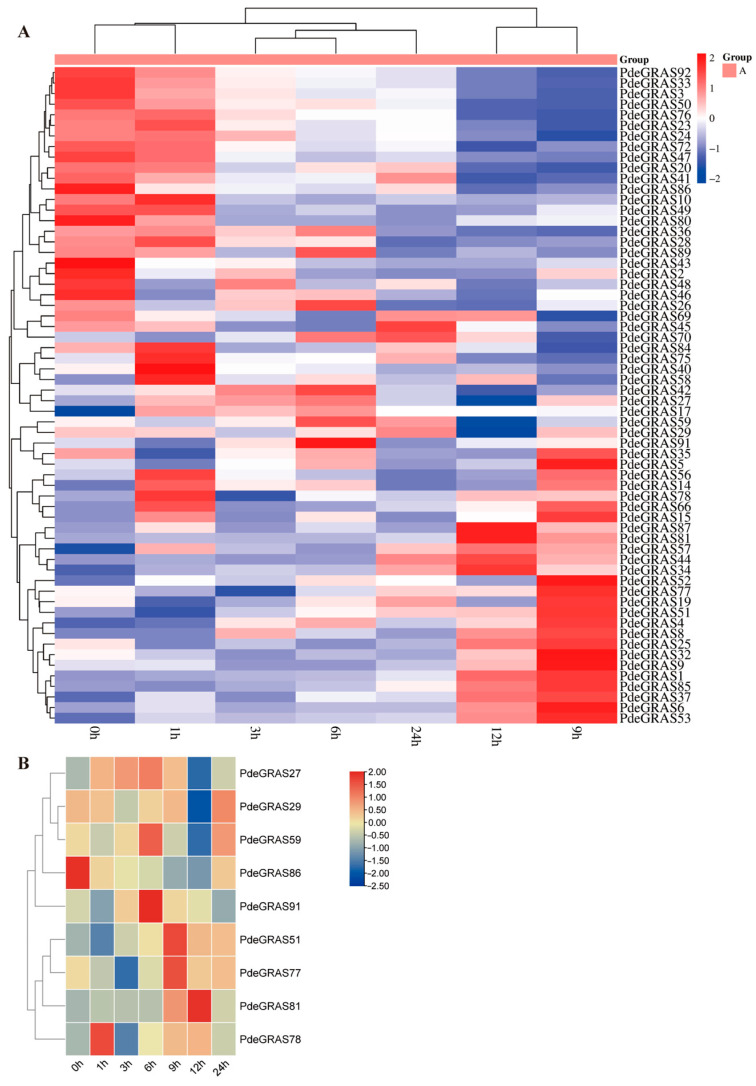
Heatmap of expression profiling of PdeGRAS family under PEG-simulated drought stress. (**A**) Expression levels of *PdeGRASs* using RNA-seq data from *P. deltoides* ‘Danhong’ seedlings exposed to 10% PEG6000-simulated drought stress at different time points. (**B**) Expression levels of *PdeDELLAs* using RNA-seq data under PEG-simulated drought stress.

To investigate the expression patterns of PdeGRAS family members under PEG-simulated drought stress, the expression levels of *PdeGRASs* were analyzed based on previous transcriptome data (Figure 5A). The results revealed that compared to the expression level of 0 h, the expression trends of the *PdeGRAS* genes vary at different time periods. For instance, genes such as *PdeGRAS27*, *PdeGRAS40*, and *PdeGRAS49* exhibited a strong response during the early stages of drought treatment (1–3 h), while *PdeGRAS51*, *PdeGRAS59*, and *PdeGRAS91* showed higher responsiveness at 6–9 h. At 12–24 h, *PdeGRAS34*, *PdeGRAS44*, and *PdeGRAS81* displayed a pronounced response. Similar situations have also been found in the gene expression patterns of the DELLA subfamily (Figure 5B). These findings suggest that different PdeGRAS members may have distinct functional roles during various phases of drought stress.

### 3.8. qRT-PCR Analysis of PdeGRAS-DELLA Genes in Response to PEG-Simulated Drought Stress

**Figure 6:**
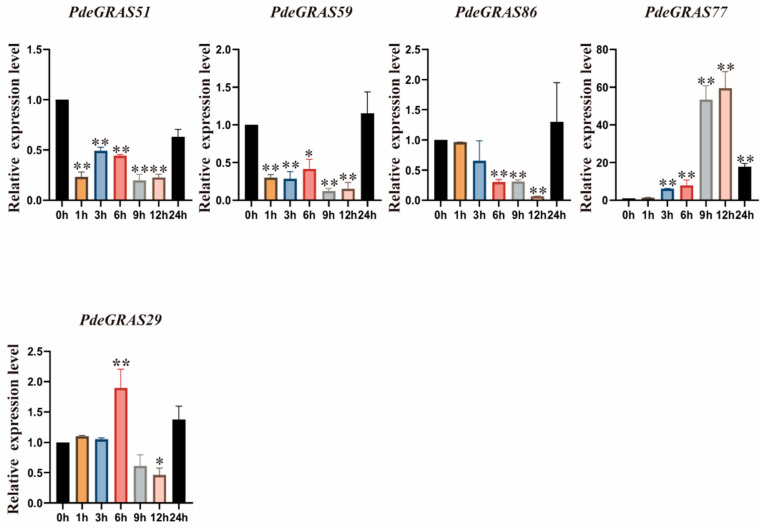
qRT-PCR analysis of five DELLA genes expression under PEG-simulated drought stress. Error bars represent the standard error of three biological replicates. Asterisks above the bars indicate statistically significant differences in expression levels relative to 0 h. * *p* < 0.05; ** *p* < 0.01.

DELLA proteins can integrate environmental signals and alter their growth in response to the surrounding environmental stresses [35]. To validate the accuracy of transcriptome-derived expression profiles, qRT-PCR was performed to quantify the expression profiles of five representative DELLA genes (*PdeGRAS29*, *PdeGRAS51*, *PdeGRAS59*, *PdeGRAS77*, *PdeGRAS86*) under PEG-simulated drought (Figure 6). The results showed that four DELLA genes exhibited expression patterns concordant with RNA-seq data, while *PdeGRAS77* displayed different expression patterns, with transcript levels significantly upregulated at the 3–24 h time points during PEG-simulated drought treatment, and the transcript level of *PdeGRAS77* peaked at 12 h. These findings suggest that *PdeGRAS77* may represent a non-canonical DELLA member, and further investigate the response of *P. deltoides* ‘Danhong’ to drought stress.

### 3.9. Protein–Protein Interaction Network and Protein Structure Prediction

**Figure 7:**
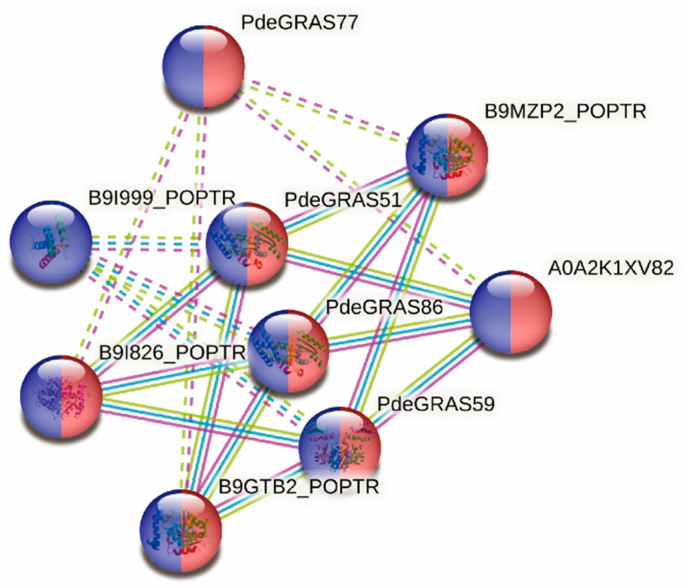
Protein–protein interaction network of PdeDELLA proteins. Network components are defined as: nodes (colored circles) denote individual proteins, connecting edges (colored lines) indicate different interaction evidence types.

To understand the protein–protein interaction (PPI) network within the DELLA subfamily, the PPI network analysis was systematically performed using the STRING database (Figure 7). Three nuclear-localized DELLA members (PdeGRAS59, PdeGRAS77, PdeGRAS86) were identified as core regulators of gibberellin (GA)-mediated signaling pathways (GO:0009938). Moreover, each exhibits transcriptional cofactor activity (GO:0003712) through conserved nuclear localization signals (Table S6). Strikingly, PdeGRAS51, PdeGRAS59, and PdeGRAS86 form a tightly interconnected triad, suggesting potential cooperative transcriptional regulation. The tertiary structures of proteins encoded by *PdeDELLA* genes were modeled using SWISS-MODEL. Five members (PdeGRAS29, PdeGRAS51, PdeGRAS59, PdeGRAS77, PdeGRAS86) exhibited conserved GRAS domain topology (Figure S2). Model reliability was confirmed by GMQE scores (PdeGRAS29: 0.74; PdeGRAS51: 0.74; PdeGRAS59: 0.75; PdeGRAS77: 0.79; PdeGRAS86: 0.73).

### 3.10. Subcellular Localization of PdeGRAS77

**Figure 8:**
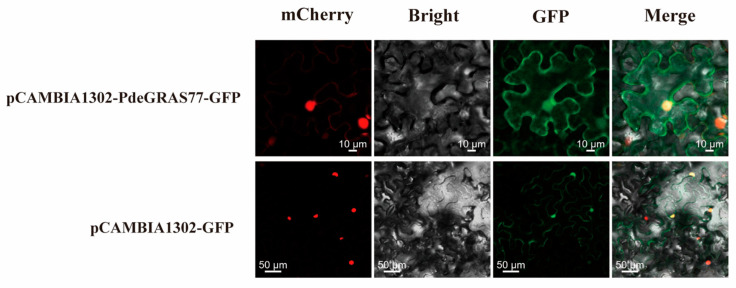
Subcellular localization of PdeGRAS77. pCAMBIA1302-GFP was empty vector, and pCAMBIA1302-35S-D53-mcherry-NOS was nucleus marker vector.

To investigate the gene function of *PdeGRAS77*, subcellular localization analysis was conducted to determine the functional locations (Figure 8). The results indicated that GFP fluorescence from PdeGRAS77::GFP was successfully expressed and was fused with the red fluorescence of the nucleus marker tagged with D53-mcherry in tobacco cells. In addition, PdeGRAS77::GFP was expressed in the cytoplasm and plasma membrane, which was not consistent with the predictions (Table S1).

## 4. Discussion

The GRAS gene family, a group of plant-specific transcription factors, has been extensively characterized across plants for its pivotal roles in developmental processes and stress responses [10,11,12]. Despite this progress, systematic investigations of GRAS family members, evolution, and functionality remain unknown in *P. deltoides*. Here, a comprehensive genome-wide characterization of the GRAS family in *P. deltoides* was performed.

Ninety-two *PdeGRAS* genes were systematically identified in the genome of *P. deltoides*. Among these, 89 *PdeGRAS* genes were unevenly distributed across 18 chromosomes with notable clustering on Chr1 (15 members) and Chr5 (8 members), while the remaining 3 genes were located on scaffolds. The physicochemical properties of the encoded proteins exhibited considerable diversity, with MWs ranging from 17.07 to 125.53 kDa and pI ranging from 4.74 to 9.63. Structural analysis revealed that *PdeGRAS* genes show high conservation in motif composition and exon–intron architecture among closely related members, which supports the functional similarity within subclades. Specially, variations in the number and position of introns within the PdeGRAS family offer valuable insights into its evolutionary trajectories, highlighting conserved and divergent genetic features that may underlie functional specialization. Phylogenetic analysis and subfamily classification revealed that GRAS members in *P. deltoides* are predominantly enriched in the LISCL (20 members), HAM (15 members), and DELLA (13 members) subfamilies. The expansion of the PdeGRAS family is predominantly driven by duplication events, including 90 segmental duplications and 11 tandem duplications. Similar duplication-driven expansion patterns have been documented in other plant species, such as soybean, *Eucalyptus grandis*, *Medicago truncatula*, and *Cunninghamia lanceolata*, where segmental duplications also dominate GRAS family evolution [36,37,38,39,40]. Notably, *P. deltoides* exhibits a two-fold expansion of GRAS members (92 genes) compared to *A. thaliana*, *Malus domestica*, *Cyclocarya paliurus* and *S. lycopersicum* [9,14,17,41]. This pronounced expansion may reflect an evolutionary strategy to enhance functional diversification, enabling adaptation to complex ecological stressors (e.g., drought, salinity) through neo-functionalization or sub-functionalization of duplicated paralogs. While the GRAS family in *Populus* species exhibits conserved subfamily architecture across angiosperms, substantial lineage-specific divergence is observed in different gene families. For instance, ClGRAS1 in ginger (*Zingiber officinale*) has been functionally linked to rhizome development, whereas PdeGRAS members show pronounced enrichment in abiotic stress pathways, reflecting functional specialization shaped by distinct evolutionary pressures [21].

*Cis*-regulatory element analysis revealed that the promoter regions of *PdeGRAS* genes were found to be enriched in regulatory motifs involved in stress-responsive and hormone-responsive elements. Promoter *cis*-element profiling in this study revealed combinatorial enrichment of ABA-, GA-, and ROS-responsive motifs across *PdeGRAS* promoters, suggesting their capacity to integrate multiple stress signaling cascades. For instance, promoters of most PdeGRASs contain ABRE motifs, ARE elements, and STRE elements. These results support the notion that there is a link between the enrichment of ABREs and the regulation of stress responses [42]. It is hypothesized that it may be involved in ABA-dependent pathways to regulate poplar drought tolerance. Future research should focus on ABA biosynthesis and signaling genes (including *PdeNCED3*, *PdeSnRK2*, *PdeRD29A*, and *PdeABI5*). The rapid-response members such as *PdeGRAS49* exhibited rapid induction during the early stress phase (1–3 h), with their promoters enriched in ABA-responsive elements (ABREs) and MYB-binding sites (MBSs). This *cis*-regulatory architecture aligns with observations in larch (*Larix kaempferi*) *LkGRAS* genes, which integrate ABA signaling and root developmental pathways to enhance drought resilience [43]. RNA-seq and qRT-PCR analysis revealed stage-specific expression patterns of PdeGRAS members under progressive drought stress. DELLA subfamily members (e.g., PdeGRAS81) exhibited significant upregulation under prolonged drought (12–24 h), suggesting their involvement in long-term stress adaptation. This functional convergence is similar to the role of SlGRAS40, a DELLA homolog in tomato (*S. lycopersicum*), which governs salt tolerance through GA-mediated regulation [17]. *PdeGRAS34* and *PdeGRAS77* also showed significant upregulation peaking at 12–24 h, implicating its involvement in long-term adaptive processes such as osmotic adjustment or cellular repair. This temporal regulatory strategy mirrors observations in *Ipomoea batatas*, where the DUF668 family member IbDUF668-6 displays phased activation during salt stress, underscoring an evolutionarily conserved mechanism of functional diversification within gene families to balance stress response efficiency and plasticity [44]. The temporal dichotomy of GRAS activation, early ABA/MYB-mediated signaling versus late GA-dependent adaptation, highlights the modular design of GRAS family in balancing immediate stress perception with sustained physiological adjustments [18].

Subcellular localization analysis PdeGRAS77 is localized to the cell membrane, cytoplasm, and nucleus, indicating that it does not exhibit the typical localization pattern of a transcription factor. Protein–protein interaction network analysis suggests that PdeGRAS77 may interact with α-β-hydrolase (ABH), an enzyme reported to be involved in regulating salt tolerance and plant development in rice [45]. Meanwhile, PdeGRAS51, PdeGRAS59, and PdeGRAS86 may also interact with bHLH transcription factors to synergistically regulate growth, development, and stress resistance in *P. deltoides*. Furthermore, this study revealed that PdeGRAS77 exhibits divergent expression patterns from other DELLA subfamily members under simulated drought stress, similar to functional homology to GA-independent DELLA signaling in tomatoes, suggesting PdeGRAS77 may operate via analogous mechanisms [46,47]. However, the molecular mechanisms underlying PdeGRAS functionality, whether through direct transcriptional regulation of downstream targets (ROS- and ABA-related target genes) or via cooperative networks with other stress-related transcription factors (e.g., MYB, NAC), remain unclear. Future investigations should employ multi-omics integration, combining post-translational modifications and CRISPR-interference screens to delineate GRAS-centered interactomes. Such approaches will bridge the gap between *cis*-element predictions and mechanistic validation, ultimately clarifying how GRAS transcription factors orchestrate stress adaptation through synergistic partnerships with MYB, NAC, and epigenetic regulators. Comprehensive analysis of these mechanisms will elucidate the evolutionary conservation and agronomic potential of GA-independent DELLA signaling in plant stress adaptation. Furthermore, this work advances our understanding of multi-gene family synergy in stress adaptation, particularly through phase-specific regulators coordinating early signaling (PdeGRAS49) and late-phase recovery (PdeGRAS77). These findings establish a mechanistic framework for engineering stress-resilient forest trees and offer a prioritized gene repository for precision breeding.

## 5. Conclusions

In this study, we conducted a genome-wide identification of GRAS transcription factors in *P. deltoides*, revealing 92 members (PdeGRASs). Through systematically identifying and characterizing their gene structures and *cis*-acting elements in promoters, as well as gene duplication, synteny, and phylogenetic relationships, we uncovered their evolutionary trajectories and putative functional divergence in drought adaptation. Transcriptomic profiling and qRT-PCR validation further revealed distinct drought-inducible expression patterns in *GRAS* genes. These findings provide a foundation for further research into the functional roles and regulatory mechanisms of *PdeGRAS* genes under drought conditions and offer a potential candidate gene, *PdeGRAS77*, for breeding drought-tolerant plant varieties.

## Data Availability

The original contributions presented in this study are included in the article/Supplementary Materials. Further inquiries can be directed to the corresponding author.

## Supplementary Materials

The following supporting information can be downloaded at https://www.mdpi.com/article/10.3390/cimb48060541/s1.
