# Supplementary material for: Genome-Wide Identification of GRAS Gene Family and Drought Response Analysis of DELLA Proteins in Populus deltoides
Source: Curr Issues Mol Biol. 2026 May 22;48(6):541. doi: 10.3390/cimb48060541 (PMC13297757; doi:10.3390/cimb48060541)
Supplement: Supplementary file 1 [file cimb-48-00541-s001.zip › cimb-4310972-supplementary-revised/cimb-4310972-supplementary-revised.pdf]

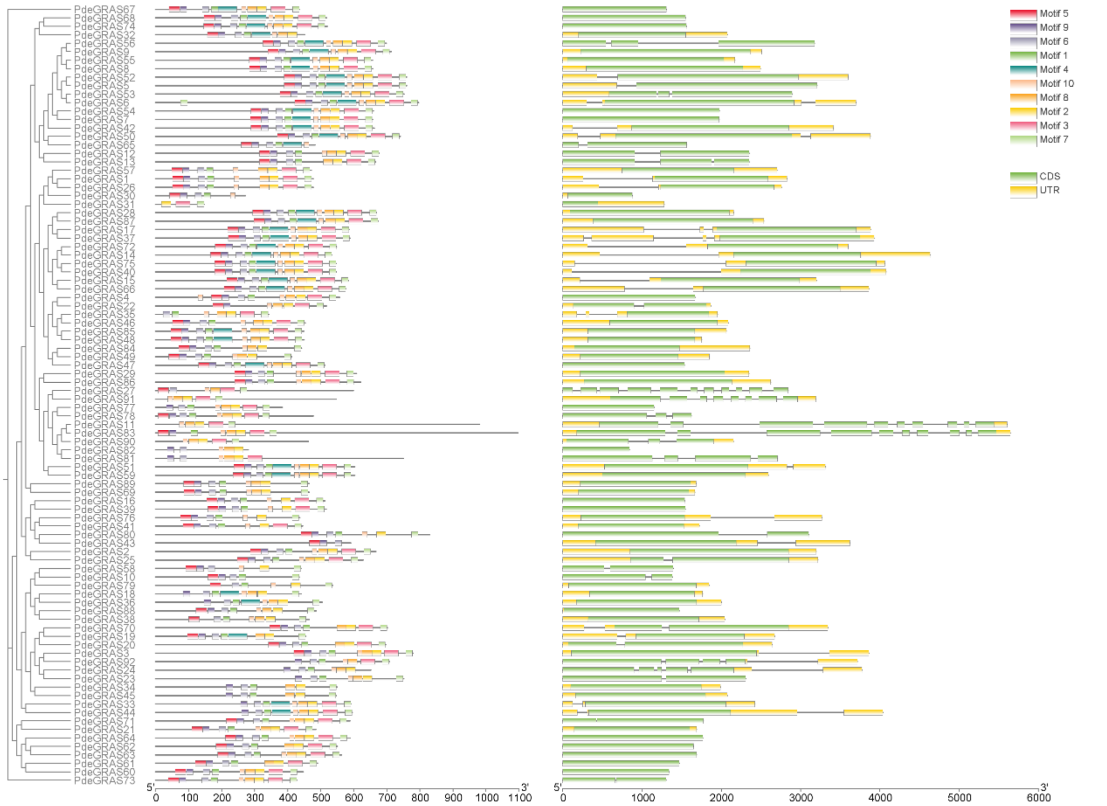

Figure S1. Gene structure and conserved motifs of the PdeGRAS family members. The horizontal axis indicates the gene and protein sequence lengths.

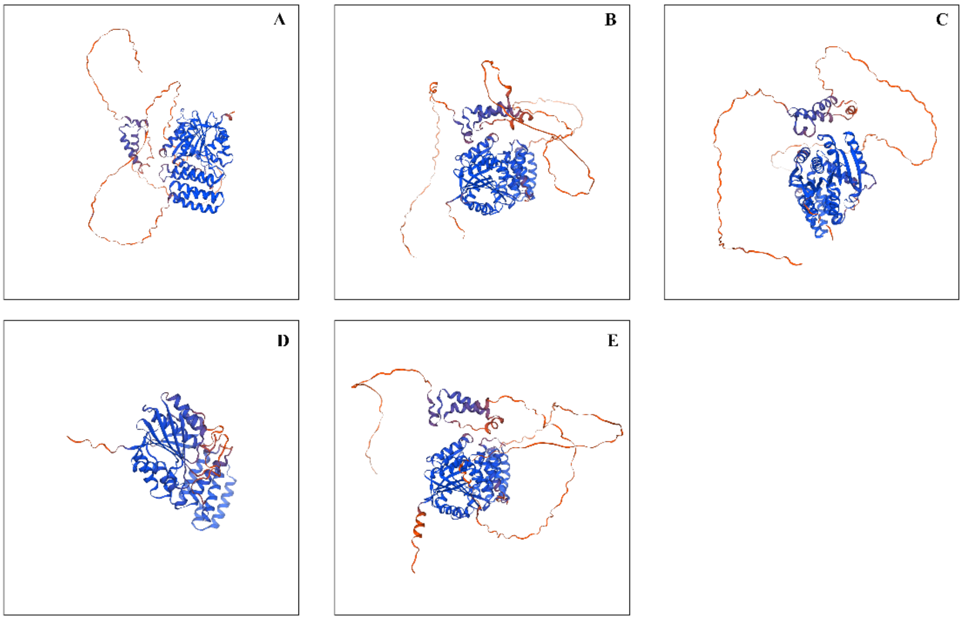

Figure S2. Three-dimensional structures of PdeDELLA proteins. (A) PdeGRAS29; (B) PdeGRAS51; (C) PdeGRAS59; (D) PdeGRAS77; (E) PdeGRAS86.
